# Self-reported cold sensitivity in normal subjects and in patients with traumatic hand injuries or hand-arm vibration syndrome

**DOI:** 10.1186/1471-2474-11-89

**Published:** 2010-05-12

**Authors:** Ingela K Carlsson, Birgitta Rosén, Lars B Dahlin

**Affiliations:** 1 Department of Hand Surgery, Skåne University Hospital, Lund University, SE-205 02 Malmö, Sweden

## Abstract

**Background:**

Cold sensitivity is a common and disabling complaint following hand injuries. The main purpose of this study was to describe self-reported consequences of cold sensitivity and the association with disability and health-related quality of life in patients with hand injuries or hand-arm vibration syndrome (HAVS) and in normal subjects.

**Methods:**

Responses to the Cold Intolerance Symptom Severity (CISS) questionnaire, Potential Work Exposure Scale (PWES), Disability of the Arm, Shoulder and Hand (DASH) and Short-Form 36 questionnaire (SF-36) were investigated in normal subjects (n = 94), hand injured patients (amputation and nerve injuries, n = 88) and patients with HAVS (n = 30). The results are presented as median (range), percent and mean deviation from norms. The Kruskal Wallis Test or Mann-Whitney U-Test were used to identify significant differences between multiple groups or subgroups. The Spearman rank correlation was used to study the relationship between cold sensitivity and disability.

**Results:**

Abnormal cold sensitivity (CISS score > 50) was seen in 75% and 45% of patients with HAVS and a traumatic hand injury, respectively. Patients were significantly more exposed to cold in their work environment than the normal population, with a consequently negative effect on work ability due to cold sensitivity. Patients with abnormal cold sensitivity were more seriously disabled and had a poorer health-related quality of life than patients with normal cold sensitivity [higher DASH scores and e.g. significantly larger mean deviation from norms in the subscales Role Physical and Bodily Pain (SF-36)].

**Conclusion:**

Severe and abnormal cold sensitivity may have a profound impact on work capacity, leisure, disability and health-related quality of life. It is frequently seen in patients with traumatic hand injuries and particularly apparent in patients with HAVS.

## Background

Cold sensitivity, described as "an exaggerated or abnormal reaction to cold exposure of the injured part, causing discomfort or the avoidance of cold", is a common complaint following hand injuries [1,2]. A variety of symptoms, such as pain, aching, numbness, weakness, stiffness and change in skin colour may be elicited by exposure to cold [3] but individual variations limit a symptom-based definition [4]. Post-traumatic cold sensitivity develops within the first few months and does not generally improve over time [2,5]. The pathophysiology behind the complex phenomenon of cold sensitivity remains unclear, thus a multifactor aetiology, including bony, vascular and neural components is suggested [6]. Cold sensitivity can occur in connection with a variety of injuries and conditions. It is frequently reported after traumatic hand injuries [7-12], after surgically treated Dupuytren's contracture [13] and in patients with hand-arm vibration syndrome (HAVS) [14,15].

Cold sensitivity may have a profound effect on health-related quality of life as well as upper extremity disability, as described in patients with specific hand injuries [16,17] and as indicated in a group of patients with digital replantation or amputation where 65% considered cold intolerance the main reason for their disability [18]. However, the impact on disability and health-related quality of life in patients with HAVS compared to patients with a traumatic hand injury and to normal subjects has not previously been considered. For a proper comparison of patient groups, validated self-report questionnaires, such as the Cold Intolerance Symptom Severity (CISS) questionnaire [3,19], are important for defining the individual patient's symptoms and signs and clarifying the impact cold sensitivity may have on daily life. Our aim, therefore was to describe the self-reported consequences of cold sensitivity and its association with self-reported disability and health-related quality of life in patients with hand injuries or HAVS and in normal subjects.

## Methods

### Study groups

During the cold season (February) of 2004 a questionnaire was sent to all patients (n = 159) with a registered diagnosis (patient register at University Hospital Malmö) of a traumatic hand injury (partial and complete amputations n = 70, major and digital nerve lesion n = 54) or HAVS (n = 35), excluding those below 18 years of age.

The patients with traumatic hand injury were treated during January-November 2003 and the patients with HAVS during 2002-2003. The patients with a traumatic injury were surgically treated at the department following the decision of the individual treating surgeon. The severity of the hand injury for each patient was defined by the hand injury severity score (HISS) [20]. Since there were no significant differences in cold sensitivity (CISS 4-100) between patients with amputation injuries and those with nerve injuries, these patients were subsumed in a single group (traumatic hand injuries). Sixteen (range 8-28) months had elapsed between the injury and the completion of the questionnaire. The diagnosis of HAVS was based on a history of vibration-induced symptoms, for example white fingers and/or sensorineural symptoms with or without impaired vibrotactile sense and neurophysiological findings supporting the presence of HAVS, but excluding other causes of neuropathy.

During the cold season (February) in 2006 the same questionnaire was sent to a normal (n = 192) random sample of subjects collected from the Swedish national population register with comparable gender (75% men, 25% woman) and age distribution (18-74 years). These subjects have previously been described with respect to the definition of a cut-off value for abnormal self-reported cold sensitivity (CISS > 50) and description of predictors of cold sensitivity [6]. One reminder was sent out to the patients and two to the normal subjects. All patients and subjects were from the southern part of Sweden which has a mean temperature of 0°C during the cold season http://www.smhi.se. A high response rate was obtained in the patient group and in the normal population. One hundred and twenty two patients (77%) (amputation n = 53, nerve-injury n = 39, HAVS n = 30) and 122 subjects (64%) from the normal population responded. No discernable differences were found between the responders and non-responders with respect to age and gender. One patient with amputation and three patients with nerve injuries were excluded due to an incorrectly registered diagnosis, leaving a total of 88 patients with traumatic hand injury and a total group of 118 patients. Twenty-eight subjects in the normal population had suffered an earlier hand injury with a consequent potential risk of cold sensitivity and were therefore excluded, leaving a total of 94 respondents. All participants gave their informed consent to participating in the study which was approved by the Ethics Committee, Faculty of Medicine, Lund University.

### Self-reported cold sensitivity, disability and health-related quality of life

Apart from the Swedish version of the CISS questionnaire (CISS score 4-100) [19], a visual analogue scale (VAS) question (0-10) concerning perceived problems on exposure to cold (0 = no problems, 10 = worst possible problems) and the Potential Work Exposure Scale (PWES) [19,21] were answered by all patients and subjects. For the patients we also included the DASH (Disability of the Arm, Shoulder and Hand) questionnaire, which contains 30 items that provide information on the patient's perception of symptoms and functional status [22], and the acute version of the Short Form 36 questionnaire (SF-36) to assess health status and health-related quality of life [23].

### Data analyses

Results are presented as median (range), percent and mean deviation from norms. The Kruskal Wallis Test was used to identify significant differences between multiple subgroups and the Mann-Whitney U-Test to analyse further significant differences between subgroups. The Spearman rank correlation was used to study the relationship between cold sensitivity and disability (DASH; r_s _> 0.30 was required). A reference material (normative data) for SF-36 with comparable gender and age distributions was distributed by the Health Related Quality of Life group in Gothenburg, Sweden http://www.hrql.se. Data were analysed using the SPSS software package, version 12.0.1.

## Results

The characteristics of the subjects and patients are described in Table 1. The patients with traumatic hand injury had a median HISS score of 75 (5-305). Twenty-five out of 30 patients with HAVS had according to the Stockholm Workshop scale vibration-induced white fingers, 27 of 30 had sensorineural symptoms and 22 of 30 had both white fingers and sensorineural symptoms (Table 1). Twenty-six of the 30 patients had an impaired vibrotactile sense, while the remaining four patients, in addition to the other criteria for HAVS had affected vibration threshold values at high frequencies. Six out of 30 patients with HAVS were operated with carpal tunnel release; however, there was no significant difference in CISS score between operated and non-operated patients (p = 0.64).

**Table 1:** Characteristics of participants.

Parameter	Normal population n = 94	Traumatic hand injury n = 88	HAVS n = 30
Gender (male/female)	69/25	69/19	26/4
Age^1^	48 (20-73)	46 (21-84)^2^	54 (24-66)^2^
Smoker Yes/No	15/79	24/66	5/25
Time since injury (months)^1^	-	16 (8-28)	-
Years of vibration exposure^1^	-	-	29 (4-46)
DASH score (0-100)^3, 1^	-	22 (1-97)^4^	38 (5-74)^4^
HISS^1, 5^	-	75 (5-305)	-
Vibration-induced white fingers (VWF)^6^	-	-	
Stage 0			13*
1			7
2			7
3			3
4			0
Sensorineural symptoms^6^	-	-	
Stage 0			3
1			9
2			10
3			8
Impaired vibrotactile sense^7^	-	-	26

^1 ^Median (range)

^2 ^Patients with HAVS were significantly older than patients with traumatic hand injuries (p = 0.01)

^3 ^0 = no disability, 100 = severest disability [22]

^4 ^Patients with HAVS had significantly higher scores indicating more severe disability (p = 0.001)

^5 ^Hand Injury Severity Score [20]

^6 ^Stockholm Workshop scale

^7 ^SI-index < 0.8 in at least one finger [35]

* 8 of 13 patients had cold sensitivity without blanching of skin (i.e. equal to 0.5 in a modified Stockholm Workshop scale, VWF)

There was an internal dropout for the CISS questionnaire (normal population, n = 13, traumatic hand injury, n = 2, HAVS, n = 2), in that not everyone answered all included questions.

### Self-reported cold sensitivity (CISS questionnaire, VAS question and PWES)

**Figure 1:**
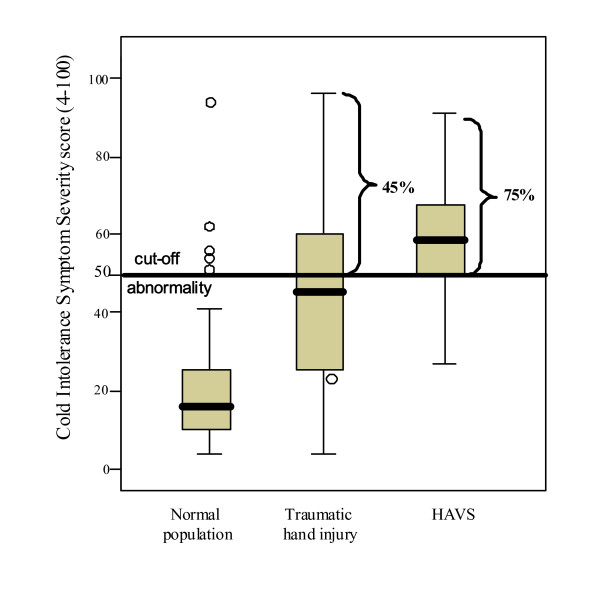
**Cold Intolerance Symptom Severity (CISS) score in subgroups**. The cut-off for abnormality is based on the 95^th ^percentile of the highest CISS scores in the normal population [6]). An abnormal CISS score was seen in 39/86 (45%) patients with a traumatic hand injury and in 21/28 (75%) of patients with HAVS. Patients with HAVS had significantly higher CISS scores than patients with traumatic hand injury (p = 0.004).

Based on a recently defined cut-off value [6] (CISS score > 50 = abnormality), abnormal cold sensitivity was seen in 4/81 (5%) of the normal population, in 39/86 (45%) of patients with traumatic hand injury [amputation injury = 26/51 (51%), nerve injury = 13/35 (37%), p = 0.208] and in 21/28 (75%) of patients with HAVS (Fig 1). Patients with HAVS had significantly higher CISS scores than patients with traumatic hand injury (p = 0.004).

**Figure 2:**
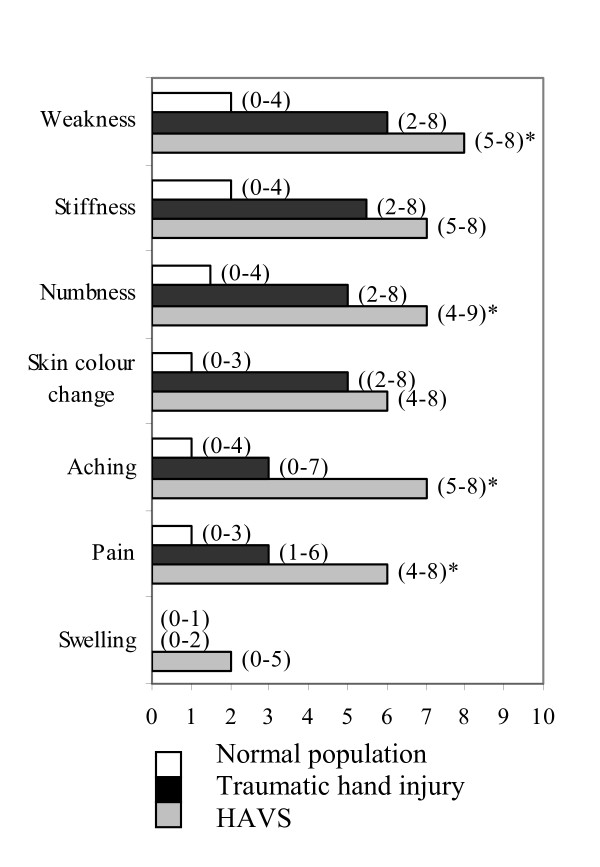
**Perceived problems on exposure to cold**. Perceived problems [bars representing median values (q_1_-q_3_)] on exposure to cold (Question 1 in the CISS questionnaire, not included in the total score) in patients with a traumatic hand injury or HAVS and in normal subjects. 0 = no symptoms/trouble at all and 10 = the most severe symptoms/trouble you can possibly imagine. *Significant differences between patients with a traumatic hand-injury and HAVS were noted for weakness (p = 0.022), numbness (p = 0.016), aching (p = 0.001) and pain (p = 0.001).

The predominant problems on exposure to cold among the patients were weakness, stiffness, numbness, skin colour change, pain and aching in their injured hands (Question no 1 in the CISS questionnaire, Fig 2). Patients with HAVS had significantly more difficult problems in their hands with weakness (p = 0.022), numbness (p = 0.016), aching and pain (p = 0.001) on exposure to cold than patients with a traumatic injury (Fig 2). Accordingly, these patients had greater problems on exposure to cold in the majority of the situations highlighted in the CISS questionnaire (Table 2). Fifty-four of the 86 patients (62%) with a traumatic injury and 24/28 patients (86%) with HAVS experienced cold-induced symptoms several times daily or continuously/all the time. The relief of symptoms on return to a warm environment varied significantly between both patient groups and the normal population (p = 0.001). It took more than 30 minutes before symptom relief was experienced in 3 of 93 subjects (3%) in the normal population, in 20/86 (23%) of patients with a traumatic hand injury and in 10/28 (36%) of patients with HAVS (Table 2).

**Table 2:** Cold Intolerance Symptom Severity questionnaire.

	**Cold Intolerance Symptom Severity questionnaire (CISS)**.	*Score*	Normal population n = 93^a^	Traumatic hand injury n = 87^b^	HAVS n = 28
	Total CISS score (4-100)	*4-100*	16 (4-62)^2^	45 (4-96)^3^	59 (23-94)^3^
1.	Not scored^1^	-	-	-	-
2.	How often do you experience these symptoms?		2 (2-10)^2^	8 (2-10)	8 (2-10)
	-continuously/all the time	*10*			
	-several times a day	*8*			
	-once a day	*6*			
	-once a week	*4*			
	-once a month or less	*2*			
3.	When you develop cold-induced symptoms, on your return to a warm environment are the symptoms relieved		2 (2-10)^2^	6 (2-10)^3^	6 (2-10)^3^
	-within a few minutes	*2*			
	-within 30 minutes	*6*			
	-after more than 30 minutes	*10*			
4.	What do you do to ease or prevent your symptoms occurring? (please tick)		4 (0-10)^2^	4 (0-10)^3^	4 (0-10)^3^
	-take no special action	*0*			
	-keep hand in pocket	*2*			
	-wear gloves in cold weather	*4*			
	-wear gloves all the time	*6*			
	-avoid cold weather/stay indoors	*8*			
	-other	*10*			
5.	How much does cold bother your injured hand in the following situations? Please score (0-10)				
	- holding a glass of ice water	*0-10*	0 (0-8)^2^	3 (0-10)	5 (0-10)
	- holding a frozen package from the freezer	*0-10*	1 (0-9)^2^	5 (0-10)^3^	7 (0-10)^3^
	- washing in cold water	*0-10*	0 (0-10)^2^	4 (0-10)^3^	7 (0-10)^3^
	- when you get out of a hot bath/shower with the air at room temperature	*0-10*	0 (0-6)^2^	0 (0-8)^3^	3 (0-10)^3^
	- during cold wintry weather	*0-10*	2 (0-10)^2^	8 (0-10)^3^	8 (4-10)^3^
6.	Please state how each of the following activities have been affected as a consequence of cold-induced symptoms in your injured hand and score each (0-4).				
	- domestic chores	*0-4*	0 (0-3)^2^	1 (0-4)^3^	2 (0-3)^3^
	- hobbies and interests	*0-4*	0 (0-4)^2^	2 (0-4)^3^	3 (0-4)^3^
	- dressing and undressing	*0-4*	0 (0-2)^2^	0 (0-4)	1 (0-4)
	- tying your shoe laces	*0-4*	0 (0-3)^2^	1 (0-4)	2 (0-4)
	- your job	*0-4*	0 (0-4)^2^	2 (0-4)^3^	3 (0-4)^3^

Median values (range) for total score and individual questions^1 ^in different subgroups.

^a and b ^Some variations in response rate (internal drop out) in separate questions were noted ^a ^n = 81-93,

^b ^n = 86-87

^1^Question number 1 concerns severity of different symptoms on exposure to cold and is not included in the total CISS score. See Fig 2 for detailed information.

^2 ^Patients (= traumatic hand injuries and HAVS subsumed together) had significantly worse scores/problems than the normal population. Mann-Whitney U- Test; p = 0.002 to 0.04

^3 ^A significant difference wasnoted between subgroups (Kruskal Wallis Test; p < 0.001). Patients with HAVS had significantly worse scores/problems than patients with traumatic hand injury. Mann-Whitney U-Test = 0.001 for all items.

Cold wintry weather was very troublesome for both patient groups in contrast to the normal subjects. Cold sensitivity also affected patients' involvement in their hobbies and interests (Table 2). Twenty-one of 39 patients (54%) with a traumatic hand injury and abnormal CISS score had severe problems with cold- induced symptoms during their leisure activities, while a clear majority (32/47, 68%) of patients with a normal CISS score only experienced minor problems or no difficulties at all.

Perceived problems on exposure to cold, expressed as median (range) scores on the VAS question, were 2 (0-9.5) for the normal subjects, 6 (0-9.8) for patients with a traumatic hand injury and 8 (1-10) for patients with HAVS. Normal subjects had significantly less problems on exposure to cold than the patients groups (p = 0.001). Patients with HAVS also had significantly greater problems than patients with a traumatic hand injury (p = 0.001).

Patients in both groups were exposed to cold in their work environment to a significantly higher degree than the normal population (PWES score, Table 3) and reported, accordingly, a negative effect on their work ability (Question 6, Table 2). Twenty-seven percent of all patients (traumatic hand injury; n = 14, HAVS; n = 9) reported that they were unable to work because of problems with cold sensitivity. Patients with a traumatic hand injury who changed their work had significantly higher CISS scores (p = 0.015) than those who remained in their previous work employment, 66 (21-86) and 45 (7-85), respectively]. There was no such difference in the patient group with HAVS [68 (27-91) and 58 (23-94), p = 0.286].

**Table 3:** Potential Work Exposure Scale (PWES).

Potential Work Exposure Scale	Normal population n = 49	Traumatic hand injury n = 51	HAVS n = 19
PWES: Total score (0-30)	5 (0-20.1)^1^	10.2 (0-25)	9.4 (0-21.6)
1. How much of your work requires manipulation of objects with your hands at temperature near or below freezing? (0-10)^2^	1.3 (0-6.4)^1^	5 (0-10)	5 (0-7.5)
2. At work, how much time are you required to be working either outside in the cold or in a refrigerated environment? (0-10)^2^	2.5 (0-7.5)^1^	5 (0-10)	5 (0-10)
3. How much do you do with the temperature at or near freezing when you are not able to wear gloves or mittens? (0-10)^2^	0.3 (0-7.5)	0.7 (0-9.3)	1.9 (0-8.7)

Median values (range) for total score and individual questions in different subgroups.

^1 ^Patients were exposed to cold in their work environment to a significantly higher degree than the normal population (p = 0.013). No significant difference was found between patients with a traumatic hand injury and HAVS.

^2 ^0 = never, 10 = all the time

### Cold sensitivity - association with disability and health-related quality of life

**Figure 3:**
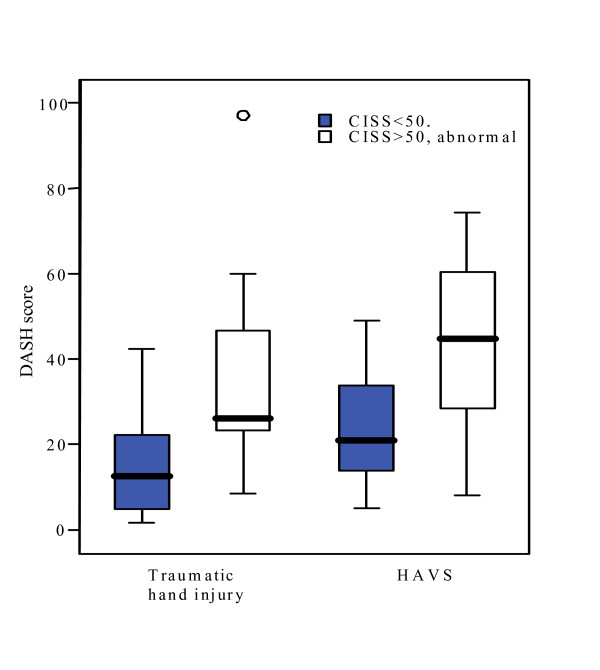
**DASH scores**. DASH scores for patients with a traumatic hand injury or HAVS divided into subgroups with normal versus abnormal CISS scores. Patients with HAVS had overall significantly higher DASH scores than patients with traumatic hand injuries (0 = 0.001). Patients with abnormal CISS scores had significantly higher DASH scores indicating more severe disability than those with normal CISS scores (p = 0.001).

The CISS score correlated significantly with the DASH score (traumatic hand injury; r_S _= 0.57, p = 0.002, HAVS; r_S _= 0.72, p = 0.001). Significantly higher DASH scores (p = 0.001), indicating more serious disability, were seen in both patient groups with abnormal CISS score compared to those with a normal CISS score (Fig 3).

**Figure 4:**
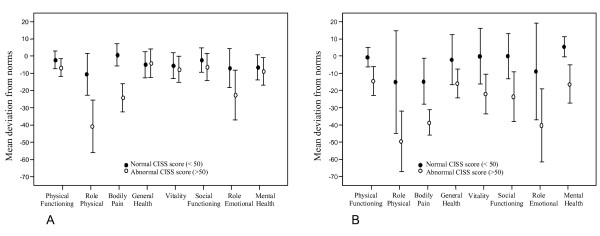
**A Health-related quality of life (SF-36) in patients with traumatic hand injury compared to normal values and B - Health-related quality of life (SF-36) in patients with HAVS compared to normal values**. A: Mean deviation from norms (95% CI) for patients with a traumatic hand injury with normal (n = 47) and abnormal (n = 39) CISS scores. Patients with abnormal CISS scores had significantly larger mean deviations from norms than patients with normal CISS scores in the subscales; Physical Functioning p = 0.041, Role Physical p = 0.003 and Bodily Pain p = 0.001, indicating poorer health-related quality of life. Normative data with comparable gender and age distribution and was distributed by the Health Related Quality of Life group in Gothenburg, Sweden http://www.hrql.se. B: Mean deviation from norms (95% CI) for patients with HAVS with normal (n = 7) and abnormal (n = 21) CISS scores. Patients with abnormal CISS scores had significantly larger mean deviations from norms than patients with normal CISS scores in the subscales; Role Physical p = 0.036, Bodily Pain p = 0.006, Vitality p = 0.03 and Mental Health p = 0.048, indicating poorer health-related quality of life. Normative data with comparable gender and age distribution and was distributed by the Health Related Quality of Life group in Gothenburg, Sweden http://www.hrql.se.

Patients with abnormal cold sensitivity (i.e. CISS score > 50) from the groups with traumatic hand injury or HAVS had a poorer health-related quality of life than the patients from these groups with normal cold sensitivity, as shown by a significantly larger mean deviation from norms in the subscales Role Physical and Bodily pain. A similar pattern of abnormality appeared for the patients with traumatic hand injury in the subscale Physical Functioning (Fig 4a) and in the subscales Vitality and Mental Health for the patients with HAVS (Fig 4b).

## Discussion

Our results show that abnormal self-reported cold sensitivity was not only observed in patients with a traumatic hand injury, but was particularly common among patients with hand-arm vibration syndrome (HAVS) as compared to normal subjects. In addition, the severity and occurrence of symptoms and the relief of cold-induced symptoms after returning to a warm environment, the influence on daily life, disability and health-related quality of life were most apparent in patients with a diagnosis of HAVS. Among our patients this diagnosis was based on a history of regular exposure to hand-held vibrating tools with white fingers on exposure to cold and/or sensorineural symptoms, an impaired vibrotactile sense and neurophysiological findings supporting HAVS but excluding other causes of neuropathy.

A novel finding in this study was the significant relationship between abnormal self-reported cold sensitivity and health-related quality of life in our patients, indicating the severe impact of cold sensitivity for the individual patient. The association was particularly apparent for subscales in the SF-36 focusing on how the magnitude and interference of bodily pain affects work performance or questions concerning satisfaction and performance at work or during other daily activities (Role Physical). In addition, abnormal cold sensitivity was also associated with greater disability, as measured using the DASH questionnaire. A more severe cold sensitivity, associated with more functional limitations and reduced health-related quality of life, has previously been reported in patients with upper extremity injuries [16]. Consequences and adaptations in daily life may also result in changed life roles and a struggle to maintain self-image [24]. A majority of vibration-exposed patients consider cold sensitivity as severe or as a problem that hinders their functioning [15]. Furthermore, they report a poorer quality of life and difficulties in the activities of daily living [25]. It is important to include an assessment of cold sensitivity for vibration-exposed patients since it may be an early neurological symptom of vibration-induced injury [14,26].

Both groups of patients had an underlying nerve injury or neuropathy. Structural changes in nerve trunks occur after a traumatic hand injury, but may also be present in HAVS [14] although possibly differing in character [27,28]. Complex structural alterations occur in nerve trunks in HAVS with demyelination, degeneration of nerve fibres of different diameter, receptor dysfunction and even a central component has recently been suggested [29,30]. Thus, the structural changes in HAVS may be even more complex, located at multiple levels, but profound changes also take place after a traumatic hand injury with nerve involvement. However, several non-neuronal factors may be predictors of cold sensitivity [6]. A difference between the two groups of patients were that patients with HAVS were older and had a history of long-time vibration exposure compared to the patients with traumatic hand injuries who were relatively recently injured. Even a short exposure time to vibrating tools may induce cold sensitivity and reduce sensitivity to temperature [31]. However, we have previously described that age peer se has no impact on cold sensitivity in neither normal's nor hand injured patients; thus, ruling out the possibility that age may explain the difference in symptoms between the groups [6]. The complex pathophysiology in HAVS may contribute to the severe self-reported cold sensitivity in this group. In addition, there was no significant difference in CISS scores between our patients with an amputation and those with a pure nerve lesion, which may indicate that nerve regeneration per se has no impact on cold sensitivity.

All patients ranked weakness, stiffness, numbness and skin-colour change as the most troublesome symptoms on exposure to cold, while pain and aching were only ranked as equally troublesome in patients with HAVS. There are some inconsistencies in previous studies concerning exactly what symptoms patients experience when exposed to cold [4,5,32]. Such inconsistencies may be explained by variations in the study population or whether the severity or presence of symptoms was evaluated. Our patients experienced cold-induced symptoms frequently with a significant difference in the time it took for patients, compared to the normal subjects, to experience relief of symptoms on return to a warm environment. Activities, such as holding a package from the freezer or washing in cold water, are problematic and cold sensitivity has a clear impact on leisure activities in a majority of patients with abnormal self-reported cold sensitivity. This is in keeping with earlier findings [24,33]. Cold, wintry weather was, as expected, very troublesome for the majority of patients. Not only temperature, but also rain, high humidity and windy weather trigger cold-induced symptoms. Patients with severe cold sensitivity may therefore also have problems during other seasons. Interestingly, seasonal variation is not included as a variable in the CISS questionnaire. One strength of our study is that we investigated the patients at a defined time-point during the cold season.

The frequent exposure to cold, as shown by high scores on the PWES, put hand-injured patients in an exposed position and forced them to change their work. Patients who had changed employment had significantly higher CISS scores than those who remained in their jobs, indicating the impact cold sensitivity may have.

### Limitations of the study

Even if we compared our patients with normal subjects the pre-existing level of cold sensitivity was unknown among our patients. We cannot, therefore, be certain how the pre-existing level of cold sensitivity may influence the level of cold sensitivity later on, after the hand trauma or vibration-exposure. We had a slight drop-out of subjects and patients although to a limited extent. A few activities included in the questionnaire did not elicit any problems for any group of patients and the validity of these items has previously been questioned (e.g. tying shoe laces, dressing and undressing). It is questionable that these items have the same impact on the total CISS score, as for example work and leisure, since they are usually performed indoors. Furthermore, Ruijs et al [34] have suggested an additional response option in questions number two (never) and number three (not applicable) to make a score of zero possible.

There was a small proportion of women in the study which corresponds to the general situation in clinical practice with respect to hand injuries and HAVS. However, we found no significant gender differences in cold sensitivity (CISS score) in any of the groups (results not shown). In addition, the ratio of male/female in our normal random sample was comparable with the group of traumatic hand injuries.

## Conclusions

In conclusion, abnormal cold sensitivity is seen in 75% of patients with HAVS and in 45% of patients with a traumatic hand injury. The predominant problems are weakness, numbness, stiffness, skin colour change and pain/aching, on exposure to cold. One important finding was a significant difference in time taken for symptoms to be relieved on return to a warm environment between both patient groups and compared to the normal population. Cold sensitivity among patients with traumatic hand injuries, such as nerveinjuries and amputations, and HAVS may have a profound impact on performance at work, in leisure pursuits and in other activities in daily life. It is apparent that there is an association between self-reported cold sensitivity and disability and health-related quality of life.

## Competing interests

The authors declare that they have no competing interests.

## Authors' contributions

IC designed the study and performed the statistical analysis. IC and LD interpreted the findings. IC, LD and BR drafted the manuscript. All authors read and approved the final manuscript.

## Pre-publication history

The pre-publication history for this paper can be accessed here:

http://www.biomedcentral.com/1471-2474/11/89/prepub
